# Overexpression of *CsHMGB* Alleviates Phytotoxicity and Propamocarb Residues in Cucumber

**DOI:** 10.3389/fpls.2020.00738

**Published:** 2020-06-12

**Authors:** Shengnan Li, Ming Xin, Jie Luan, Dong Liu, Chunhua Wang, Chunhong Liu, Wenshuo Zhang, Xiuyan Zhou, Zhiwei Qin

**Affiliations:** ^1^College of Horticulture and Landscape Architecture, Key Laboratory of Biology and Genetic Improvement of Horticultural Crops (Northeast Region), Northeast Agricultural University, Harbin, China; ^2^College of Horticulture, Fujian Provincial Key Laboratory of Haixia Applied Plant Systems Biology, Fujian Agriculture and Forestry University, Fuzhou, China

**Keywords:** cucumber, propamocarb residues, *CsHMGB*, antioxidant enzymes, AsA-GSH system

## Abstract

Cucumber (*Cucumis sativus* L.) is one of the most economically important fruits of the Cucurbitaceae family, therefore consideration of potential pesticide residues in the fruit in the context of cucumber breeding and production programs is important. Propamocarb (a pesticide commonly used to prevent downy mildew) is widely used in cucumber cultivation, but the molecular mechanism underlying the degradation and metabolism of propamocarb in cucumber is not well understood. We screened a candidate *CsHMGB* gene (*CsaV3-5G28190*) for response to propamocarb exposure using transcriptome data. The coding region of *CsHMGB* was 624 bp in length and encoded the conserved HMB-box region. *CsHMGB* expression differed significantly between the “D0351” genotype, which accumulated low levels of propamocarb, and the “D9320” genotype, which accumulated high levels of propamocarb. *CsHMGB* expression was positively correlated with propamocarb levels in the cucumber peel. *CsHMGB* expression was upregulated in the fruit peels of the “D0351” genotype following exposure to propamocarb stress for 3–120 h, but no difference was observed in expression between propamocarb treatment and control for the “D9320” genotype. For the “D0351” genotype, *CsHMGB* expression was higher in the fruit peels and leaves than that in female flowers; expression was moderate in the stems and fruit pulps, and weak in male flowers and roots. The CsHMGB protein was targeted to the nucleus in *Arabidopsis* protoplasts and in the epidermis of *Nicotiana benthamiana* leaves. We measured MDA, O_2_^–^, and H_2_O_2_ levels in cucumber plants and found that they were likely to accumulate reactive oxygen species (ROS) in response to propamocarb stress. Analysis of antioxidant enzyme activity (SOD, POD, CAT, APX, GPX, GST, and GR) and the ascorbate-glutathione (AsA-GSH) system showed that the resistance of the plants was reduced and the levels of propamocarb residue was increased in CsHMGB-silenced plants in response to propamocarb stress. Conversely, overexpression of *CsHMGB* promoted glutathione-dependent detoxification by AsA-GSH system and improved the antioxidant potential, reduced the accumulation of ROS. Ultimately, the metabolism of propamocarb in cucumber was increased via increase in the wax levels and the stomatal conductance.

## Introduction

Cucumber (*Cucumis sativus* L.) is one of the most economically important fruits in the Cucurbitaceae family and is widely cultivated in greenhouses, where it is susceptible to downy mildew (Liu et al., 2017). Propamocarb is a carbamate fungicide that is used to control downy mildew (Sharma et al., 2016). Most pesticides are heterologous toxic substances, and excessive pesticide application can result in phytotoxicity, increased cell membrane permeability, impaired metabolism, and declined quality and yield (Zhou et al., 2015). Excessive use of pesticides in cultivation leads to lingering residues in cucumber fruit, and can contaminate the fruit and the surrounding environment. Pesticide residue must therefore be carefully considered in the context of cucumber breeding and production programs.

We have previously shown that the magnitude of propamocarb residue accumulation in cucumber is a polygene-regulated quantitative trait. In addition, different genotypes of cucumber differ significantly with respect to residue accumulation (Ma, 2010). Genes involved in propamocarb stress were identified in cucumber through transcriptome analysis, and we screened a candidate high-mobility-group gene (*CsHMGB*) which was upregulated in response to propamocarb stress (Wu et al., 2013). Based on gene function, we speculated that *CsHMGB* may be crucial in mediating the response to propamocarb stress.

High-mobility-group (HMG) protein is a highly abundant non-histone chromosomal protein that is found in eukaryotes. Human diseases, cell damage, trauma-induced stress and necrosis, chronic inflammation, autoimmune diseases, and cancer may all lead to the release of the HMGB1 protein. Extracellular HMGB1 stimulates the inflammatory response and recruits leukocytes by interacting with receptors for advanced glycation end-products (RAGE), toll-like receptor 2 (TLR), and other receptors to protect the organism from disease (Yang et al., 2010; Harris et al., 2012; Venereau et al., 2016). In plants, HMG proteins can be divided into four categories based on their structural characteristics: HMGB type (proHMG-1/-2) protein, containing a characteristic HMG-box domain; ARID-HMG protein, characterized by an A/T-rich binding domain; 3 × HMG protein, containing three HMG-boxes; and SSRP1, a structure-specific recognition protein (Antosch et al., 2012). The HMGB protein is a chromatin structure regulator involved in a variety of stress-tolerance mechanisms in plants. In *Arabidopsis thaliana*, *AtHMGB2* and *AtHMGB3* were significantly downregulated in response to drought and salt stress, but were upregulated in response to cold stress (Kwak et al., 2007; Lildballe et al., 2008). *AtHMGB11* is a nuclear protein that regulates chromatin dynamics and transcription. In *Arabidospsis thaliana*, it interacts with the major groove of other proteins in response to drought stress (Adrita et al., 2016). *AtHMGB15* interacts with the pollen transcription factors, *AtAGL66* and *AtAGL104* to alter pollen germination and growth of the pollen tube (Xia et al., 2014). *AtHMGB3* has been shown to induce the transcription of *AtWRKY33* and *AtFDF1.2*, and to increase the accumulation of corpus callosum (Choi et al., 2016). The HMGB proteins also interact with other proteins, such as histones and transcription factors (Grasser et al., 2007). However, the function of *CsHMGB* in response to propamocarb stress has not been reported in cucumbers.

The detoxification of pesticides [herbicides and chlorothalonil (CHT)] in plants can be divided into four phases: (1) hydrolysis of peroxidase and cytochrome P450s; (2) catalysis of glutathione-*S*-transferase (GST) and glycosyltransferase; (3) transmembrane transport of transporters such as the ABC family; and (4) the hydrolase degradation reaction. The first two steps degrade toxic macromolecular groups into lower levels of toxicity and create poor mobility metabolites, and the latter two steps are responsible for the further hydrolysis and transport of low-toxicity small molecular compounds (Muntane, 2009; Zhou et al., 2015). Additional self-protection mechanisms in plants such as the presence of wax and stomatal action are essential for resisting pesticide stress. In a previous study, we found that the increased opening of stomatal apertures contributes to low levels of pesticide residue (Zhao, 2013). Pesticides can be lost through the stomata during transpiration (Yao and Yang, 2012). Wax is a natural protective screen against plant diseases, insect pests, and external stresses (Yeats and Rose, 2013; Bohinc et al., 2014).

To investigate the mechanisms underlying propamocarb residue accumulation in cucumbers, we identified a putative gene, *CsHMGB*, involved in mediating propamocarb stress. Functional analyses revealed that overexpression of *CsHMGB* could alleviate propamocarb phytotoxicity by increasing the waxiness of the cuticle, increasing the stomatal conductance in the pericarp cells, and activating the antioxidant system.

## Materials and Methods

### Plant Materials

Two cucumber genotypes exhibiting different levels of propamocarb residues in the fruit peel were used: “D0351” (low accumulation of propamocarb residue; 0.014 mg/kg) and “D9320” (high accumulation of propamocarb residue; 0.171 mg/kg). For each genotype, 200 plants were sown in pots containing a mixture of soil and vermiculite (v:v = 1:1) in a solar greenhouse at the Northeast Agricultural University (Harbin, China) (Wu et al., 2013). Plants were grown under a 12 h photoperiod at 28°C/18°C (day/night) and 75% relative humidity.

### Propamocarb Treatments

To investigate phytotoxicity in cucumber plants, we sprayed 20 μL propamocarb (400, 800, and 1,200 ppm) onto the leaves of “D0351” and “D9320” plants. Leaves of similar size were selected at 48 h after propamocarb application for further study. MDA content was measured by reacting MDA with thiobarbituric acid (TBA), and MDA-TBA adduct formation was measured based on absorbance at 450, 532, and 600 nm wavelengths (Foyer and Halliwell, 1976).

To determine the accumulation of propamocarb residues in various organs, we applied 50 mL propamocarb (400 ppm) to the entire plant until liquid dripped from the leaves and fruits at 34 days after planting; an equivalent volume of water was applied as a control. Each plant was sprayed once. Samples were collected from five individual plants at 6, 9, 12, 24, 36, 48, 72, 96, and 120 h after propamocarb application. Fruit were collected from the 10th node. Fruits with the following parameters were selected: length 16–18 cm, diameter 5–6 cm, and weight 160–180 g (Liu et al., 2018; Zhang et al., 2019b). Fruit peels were cut into 2 mm thick samples and fruit pulp sections were cut to 5-mm thickness. Stems and leaves connected to fruits were collected, and female and male flowers in bloom were also collected (Supplementary Figure S1). Roots were collected, cleaned, and dried (Liu et al., 2018).

The “D0351” genotype was used for the gene silencing experiment and was planted in an artificial climate incubator (28/18°C, day/night).

The “D9320” genotype was utilized for overexpression studies. T_1_ lines of OX2, OX3, and OX5 (each line including five individual plants per biological repeat; three biological repeats) were sown in growth chambers. Plants were treated with propamocarb, and the samples were harvested as previously described.

### Determination of Propamocarb Residues

A propamocarb analytical standard was provided by Sigma-Aldrich^®^ (CAS: 24579-73-5) (Merck, United States). Ten grams of homogenized sample were added to a 50 mL colorimetric tube and further homogenized for 5 min. Then, 20 mL acetonitrile and 2.5 g NaCl were added to the sample in the 50 mL colorimetric tube, and mixed for 5 min with shaking at 500 g/min using a mechanical shaker. Samples were allowed to settle for 30 min and then vortex mixed and evaporated until dryness; this step was repeated several times and the resulting material was collected. The sample was then filtered through a polypropylene filter (Merck, United States) (0.22 μm) until no residual particles remained. The levels of propamocarb residue were then analyzed using the Agilent 7890B-5977A gas chromatography system (Agilent Technologies) with a 30 m × 0.25 mm × 0.25 μm capillary column (HP-5MS). The oven temperature was programmed at 40°C 2 min, increased to 200°C at 25°C/min and held for 8 min, sample inlet: 240°C, non-split injection, flow rate of 1.0 mL min^–1^.

### *CsHMGB* Cloning and Bioinformatic Analysis

The coding sequence for the full-length *CsHMGB* gene was acquired from the cucumber genome database^1^. Fruit peels from cucumber genotype “D9320” were used to clone *CsHMGB* with specific primers (Supplementary Table S1). Polymerase chain reactions were performed using the following protocol: 94°C for 2 min; 30 cycles of 94°C for 10 s, 56°C for 15 s, and 72°C for 10 s; and a final extension at 72°C for 5 min. HMGB protein sequences from other species were obtained from the NCBI database^2^. DNAMAN software^3^ was used for multiple sequence alignments. MEGA 7.0 software (Poisson model and bootstrap = 1,000) was used to construct a neighbor-joining (NJ) phylogenetic tree (Kumar et al., 2016).

### First-Strand cDNA Synthesis and qRT-PCR Assays

Total RNA was isolated from fruit samples using TRIzol reagent (Invitrogen, Carlsbad, CA, United States). This RNA was then used to synthesize single-stranded cDNA using a reverse transcription kit (Toyobo, Japan), And qRT-PCR was performed in a 20 μL reaction containing 2× Fast qPCR Master Mix (DiNing, China; 10 μL), cDNA template (2 μL), forward and reverse primers (0.5 μL; 10 μmol L^–1^), and ddH_2_O. Cucumber *EF1a* was used as an internal reference gene to calculate the relative expression of the candidate genes (Wan et al., 2010) using the 2^–ΔΔ*CT*^ method (Schmittgen and Livak, 2008). The program was run as follows: 95°C for 2 min, then 40 cycles of 95°C for 15 s, 56°C for 15 s, and 72°C for 30 s. The melting curve was generated as per a default application on qTower2.0 (Analytikjena, Germany). All primers used for qRT-PCR are shown in Supplementary Table S1. Three biological replicates and three technical replicates were performed.

### Subcellular Localization

The full-length sequence of *CsHMGB* (with the exception of TAG) was amplified using PCR. *CsHMGB* was cloned into a green fluorescent protein (GFP) carrier with a super promoter (pSuper-1300) via traditional digestion (*Xba*I and *Bgl*II) and T4 ligation (Invitrogen, Carlsbad, CA, United States) (Li et al., 2018). The empty vector was used as a negative control. Following this process, *A. thaliana* protoplasts were transfected with recombinant CsHMGB-GFP and the empty GFP vector, and were imaged using a confocal spectral microscope with green fluorescence at 488 and 580 nm (Yoo et al., 2007) (Leica, Germany).

The CsHMGB-GFP and empty GFP plasmids were transferred into *Agrobacterium tumefaciens* GV3101 using the freeze-thaw method, and the leaves of 5-week-old *Nicotiana benthamiana* plants were inoculated with the *A. tumefaciens*. The tomato bushy stunt virus gene (*p19*) suppresses the silencing of *CsHMGB* (Grefen et al., 2008). After 2 days, leaf epidermal cells from *N. benthamiana* were observed using a confocal microscopy, as described above.

### Silencing of *CsHMGB*

Virus-induced gene silencing (VIGS) for *CsHMGB* was performed in cucumber leaves at 14 days after the seeds were sown (at one-leaf stage). A specific 248 bp fragment of *CsHMGB* was amplified and then cloned into the pTRV2 vector using *Ecor*I with *Bam*HI. Plasmids were used to transform *A. tumefaciens* strain GV3101, which were then cultivated in LB medium containing 50 μg mL^–1^ rifampicin and 50 μg mL^–1^ kanamycin on a rocking platform at 200 rpm and at 28°C for 18 h. Cells were collected and re-suspended in an infiltration buffer supplemented with 200 mM acetosyringone, 10 mM MgCl_2_, and 10 mM MES (pH 5.6), at a final OD_600_ of approximately 1.2. Culture mixtures primarily contained pTRV2-*CsHMGB* and pTRV1-*CsHMGB* (v/v, 1:1), or pTRV2 and pTRV1 (negative controls), and were incubated for 3–4 h under 25°C in darkness prior to injection. Silencing of the plant alkene desaturase (PDS) gene causes photobleaching, which can be used to control the efficiency of VIGS (André et al., 2009). In total, there were 30 plants in each combination, which were divided into three groups of 10 plants as biological repeats. Three biological and technical replications were performed.

### Cucumber Transformation System

The amplified *CsHMGB* sequence was cloned into the pCXSN-1250 vector harboring the glyphosate resistance gene under a constitutive promoter (Chen et al., 2009). Digestion was performed with *Xcm*I, following which the method followed that described for the TA clone system; the vector was ligated with the *CsHMGB* PCR products using T_4_ DNA ligase (Invitrogen, Carlsbad, CA, United States), thus generating the pCXSN-1250-*CsHMGB* overexpression vector. Agrobacteria from the GV3101-mediated method were used to infect the cotyledons of cucumber plants to generate transgenic plants. Cells with over-expression were selected in MS medium supplemented with 1 mg/L glufosinate (Zhang et al., 2014). The generation of T_0_ and T_1_ cucumber plants was verified via PCR and qRT-PCR. Primer sequences are displayed in Supplementary Table S1.

### Analysis of Pericarp Cells

The exocarp tissue in the middle of the cucumber fruit peel was sectioned into 2 × 5 mm strips with a double-sided blade. The tissue was then immediately fixed in 2.5% glutaraldehyde (phosphate buffer), immersed in the solution by vacuum pumping, and stored at 4°C for 1.5 h. Samples were then flushed, dehydrated, replaced, and freeze-dried. Conductive tape was glued to the scanning electron microscope table, and the sample surface was coated with a 15 nm metal film using an Emur1010 (HITACHI) ion sputtering coating instrument. Samples were observed and imaged under a Smur3400N scanning electron microscope.

### Reactive Oxygen Species Accumulation and Enzymatic Activity Analyses

Samples were collected from five individual plants at 2, 4, 6, 8, and 10 days after propamocarb application (Zhang et al., 2019b). Leaves connected to fruits (approximately 0.5 g leaf weight) were collected in order to measure the physiological index for the leaf tissue.

H_2_O_2_ content was measured by sulfuric acid peptide oxidation at a 415 nm characteristic absorption. The 0.5 g sample was grinded in the 2 mL pre-cooled acetone in an ice bath followed by centrifugation for 20 min at 10,000 *g* at 4°C, the supernatant was brought to 3 mL. Then, 1 mL extracting solution, 0.1 mL 20% titanic tetrachloride and 0.2 mL concentrated NH_4_OH was mixed for 5 min and centrifuged for 15 min at 10,000 *g* at 4°C. The supernatant was discarded and the precipitate was dissolved in 1 mol L^–1^ H_2_SO_4_, and washed several times with cold acetone, the final volume was made up to 2 mL (Gong et al., 2013).

O_2_^–^ content was assayed using hydroxylamine hydrochloride at 530 nm. The 0.5 g sample added 2 mL phosphoric acid buffer (50 mol L^–1^, PH = 7.80) with full grinding, and collecting the supernatant after centrifugation at 10,000 *g* for 20 min, then the volume was adjusted to 3 mL. The 0.5 mL extracting solution mixed with 0.5 mL phosphoric acid buffer (50 mol L^–1^, PH = 7.8) and 1.5 mL hydroxylamine hydrochloride (1 mol L^–1^), placed at 25°C for 1 h. Then, addition with 2 mL p-aminobenzene sulfonic acid (17 mol L^–1^) and 2 mL α-naphthylamine (7 mol L^–1^), the mixture was incubated at 25°C for 20 min (Gong et al., 2013).

The 0.5 g sample was ground to a homogenate with 3 mL pre-cooled potassium phosphate buffer (50 mol L^–1^, PH = 7.0) in an ice bath followed by centrifugation for 20 min at 10,000 g at 4°C. Extracting solution was used to determine the SOD, POD, CAT, and APX activity. POD activity was determined via the catalytic H_2_O_2_ oxidation of specific substrates by the guaiacol method at 470 nm (Bradford, 1976). SOD activity was examined by measuring the ability of SOD to inhibit the photochemical reduction of nitro blue tetrazolium at 560 nm (Aebi, 1984). CAT activity was determined via the reduction at 240 nm for H_2_O_2_ extinction (Giannakoula et al., 2010). APX activity was measured based on the rate of ASA oxidation. 0.1 mL supernatant was mixed with 1 mL extracting solution (50 mol L^–1^ potassium phosphate with pH = 7.0, 750 μmol L^–1^ AsA and 100 mol L^–1^ H_2_O_2_). The absorbance at 290 nm was measured at every 15 s intervals for 3 min (Durner and Klessig, 1995).

GPX activity was assayed through the oxidation of glutathione by GPX at the absorbance of 412 nm. The 0.5 g sample was ground to a homogenate with 5 mL extracting solution in an ice bath followed by centrifugation for 10 min at 10,000 *g* at 4°C (Anderson and Davis, 2004).

The oxidation rate of ASA measured at 265 nm was used to estimate ASA content. The 0.5 g sample was ground to a homogenate with 5 mL buffer I in an ice bath followed by centrifugation for 20 min at 8,000 *g* at 4°C. A total of 20 μL supernatant was mixed with 160 μL buffer II and 20 μL buffer III from the kit (Solarbio, Beijing). The absorbance at 290 nm was measured at 10 and 130 s; DHA content was calculated from the rate of AsA formation at a wavelength of 265 nm at 10 and 130 s (Solarbio, Beijing).

DHAR and MDHAR activity were analyzed as described by de Pinto et al. (1999). The 0.5 g sample was grinded in the pre-cooled 50 mol L^–1^ phosphoric acid buffer (containing 1 mol L^–1^ EDTA, 2.5% Triton X-100,1 mol L^–1^ascorbate, and 2% polyvinylpyrolidone) in an ice bath followed by centrifugation for 20 min at 16,000 *g* at 4°C. A total of 0.3 mL extracting solution with 50 mol L^–1^ phosphoric acid buffer (pH = 7.0), 20 mol L^–1^ GSH and 2 mmol L^–1^ DHA up to 3 mL was used to measure DHAR activity at 265 nm. A total of 0.09 mL extracting solution with 50 mol L^–1^ phosphoric acid buffer (pH = 7.0, containing 2 mmol L^–1^ AsA), 2 mmol L^–1^ NADPH and 2 U AAO (pH = 5.6) was used to measure MDHAR activity at 340 nm.

Total glutathione and GSSG content were analyzed as described by Anderson et al. (1992). The 0.5 g sample was ground to a homogenate with 2.5 mL 7% pre-cooled sulfosalicylic acid in an ice bath followed by centrifugation for 20 min at 16,000 g at 4°C. A total of 0.1 mL supernatant was mixed with 0.2 mol L^–1^ PBS, 0.2 mol L^–1^ NADPH and 1 U GR up to 3 mL. The mixture was incubated at 27°C for 30 min and the absorbance was at 412 nm measured for total glutathione content. The difference between the determination of GSSG and total glutathione was the reaction system containing 0.1 mol L^–1^ 2-vinylpyridine, and the mixture was incubated at 27°C for 1 h for determination of GSSG content. The GSH content was then calculated by subtracting GSSG from total glutathione.

Glutathione-*S*-transferase activity was measured spectrophotometrically using 1-chloro-2,4-dinitrobenzene (CDNB) and 5 mM GSH as standard substrates at a wavelength of 340 nm. The 0.5 g sample was ground to a homogenate with 5 mL 50 mol L^–1^ phosphoric acid buffer (1 mol L^–1^ EDTA, 10 mol L^–1^ KCl, 5 mol L^–1^ DTT, 0.5 mol L^–1^ AEBSF, and PH = 7.5) and polyvinyl-polypyrrolidone (insoluble PVPP), followed by centrifugation for 20 min at 12,000 *g* at 4°C (Anderson and Davis, 2004).

GR activity was detected via the estimation of the dehydrogenation rate of NADPH. The 0.5 g sample was grinded in the reaction mixture (0.1 mol L^–1^ EDTA, 3% Triton X-100, and 50 mol L^–1^ phosphoric acid buffer with 4% polyvinylpyrolidone and PH = 7.5), the volume was adjusted to 4 mL followed by centrifugation for 15 min at 16,000 *g* at 4°C. A total of 0.1 mL supernatant was mixed with 0.1 mM Tris-HCl (PH = 8.0), 1 mol L^–1^ GSSG and 0.5 mM NADPH up to 3 mL with a characteristic absorption peak at 340 nm (Anderson and Davis, 2004).

### Statistical Analysis

The statistical significance of differences was calculated by Tukey’s tests using Statistical Analysis System (SAS) version 9.21. Data are means (±SE) of three independent experiments each corresponding to at least three replicates. Statistically significant differences were determined by Student’s *t* tests (*p* < 0.05 significance level and *p* < 0.001 extremely significant level).

## Results

### Phytotoxicity of Propamocarb in Cucumber

**FIGURE 1:**
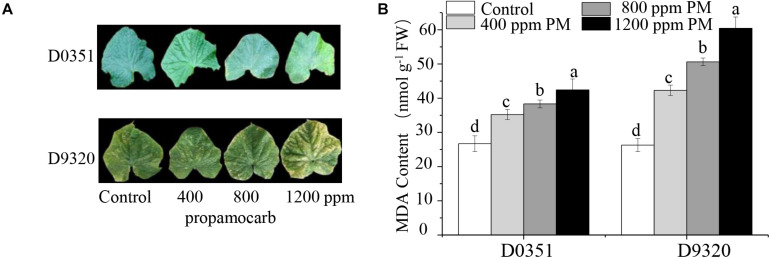
Effects of propamocarb application in cucumbers. **(A)** Phenotype of cucumber leaves following 400, 800, and 1,200 ppm propamocarb applied to “D0351” and “D9320” genotype plants at 2 days after application. **(B)** MDA content in “D0351” and “D9320” genotype plants at 2 days. Data are the means of three replicates with SEs, and different letters indicate a significant difference (*p* < 0.05 by Tukey’s test).

To determine the phytotoxicity of propamocarb in cucumber, we applied three different concentrations of propamocarb (400, 800, and 1,200 ppm) to the leaves of the “D0351” and “D9320” genotypes. The recommended dose of 400 ppm did not induce phenotypic changes in cucumber. However, excessive use of propamocarb (800 and 1,200 ppm) could induce phytotoxicity, such as yellow speckles on leaves (Figure 1A). MDA content was correlated with propamocarb concentration in both of the cucumber genotypes. MDA content was higher in “D9320” than that in “D0351” plants following propamocarb application (Figure 1B), indicating that the antioxidative capacity of the cucumber plants was reduced under propamocarb stress.

### Propamocarb Residues in Cucumber

**FIGURE 2:**
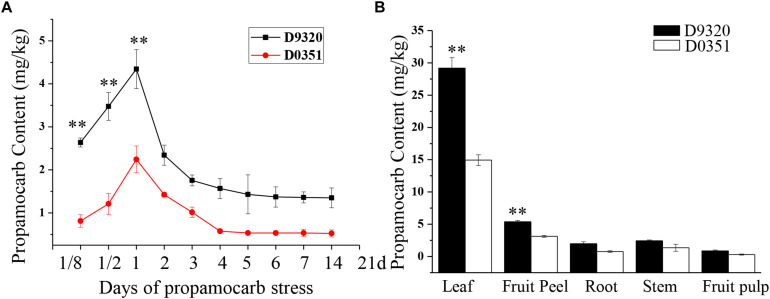
Propamocarb residues in cucumbers. **(A)** Time course of propamocarb residue in cucumber fruit peels of “D0351” and “D9320” plants. **(B)** Propamocarb residues in leaf, stem, root, fruit peel, and fruit pulp of “‘D0351” and “D9320.” Data are means (±SE) of three independent experiments with three replicates for each. Statistically significant differences were determined by Student’s *t*-test (*p* < 0.05).

We detected the propamocarb residues in “D0351” and “D9320” genotype, results showed that the maximum residue in found in the fruit peels was 2.08-fold higher in “D9320” than that in “D0351” after 2 days of treatment with 400 ppm propamocarb. Dramatic changes in fruit peel propamocarb residue were noted in both “D0351” and “D9320” after application for 3 h to 21 days. Propamocarb residues gradually increased from 12 h to 2 days of propamocarb application and dramatically decreased from 2 to 5 days. However, propamocarb residues rarely decreased between 5 and 21 days of propamocarb application (Figure 2A). Detection of propamocarb residues in various tissues revealed that residues in leaves were greater than in fruit peels, and that there were limited residues in the roots, stems, and fruit pulps (Figure 2B).

### Identification and Cloning of *CsHMGB*

As compared to water as a control, *CsaV3-5G28190* expression significantly changed under propamocarb stress, and the calculated transcript level was consistent with qRT-PCR results. The full-length *CsHMGB* cDNA sequence was cloned through RT-PCR based on the “D0351” genotype total RNA. That genomic coding region was 624 bp in length, including eight exons and seven introns with the start codon of ATG to TAA. The amino acid sequence of *CsHMGB* was obtained from cucumber plants, HMGB proteins were obtained from other Cucurbitaceae crops, and HMGB1/2/7 was obtained from *Arabidopsis* and other plant species. The results indicated that *CsHMGB* from cucumber, CmHMGB7 from melon (*Cucumis melo* L.), MnHMGB7 from bitter gourd (*Momordica charantia* L.), and CpHMGB7 from pepper (*Capsicum annuum* L.) were in the same evolutionary branch, as their genetic relationships were greater than 80%. The alignment of amino acid sequences showed that the *CsHMGB* protein contained the HMB-box conserved region (Supplementary Figure S2), and it was therefore placed in the HMGB protein family.

### Expression of *CsHMGB* Exhibits Positive Correlation With Propamocarb Residue in Cucumber Fruit

**FIGURE 3:**
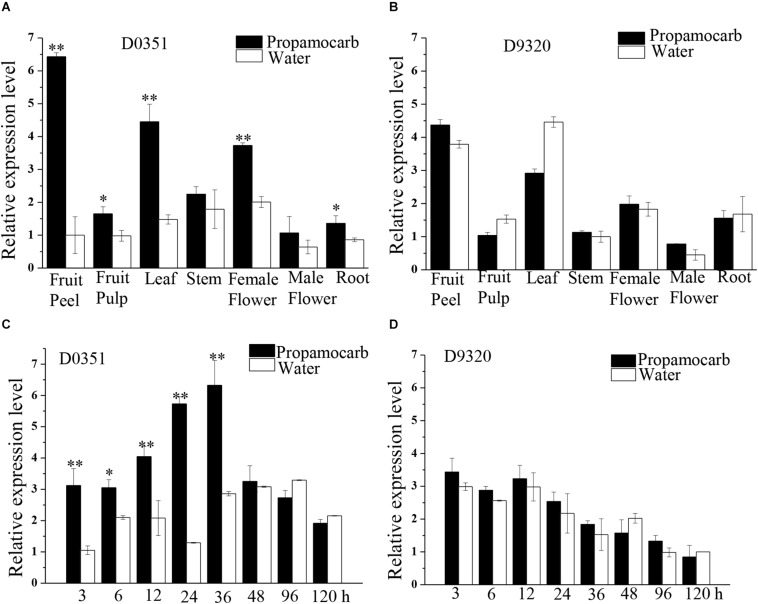
Expression patterns of *CsHMGB* in cucumbers. **(A)** *CsHMGB* levels in various tissues of “D0351” plants under propamocarb stress at 2days after application. **(B)** *CsHMGB* levels in different tissues of “D9320” plants under propamocarb stress at 2 days. **(C)** Time course of *CsHMGB* levels in fruit peels following propamocarb stress in “D0351” plants. **(D)** Time course of *CsHMGB* levels in fruit peels following propamocarb stress in “D9320” plants. *CsEF1a* was used as the reference gene. Significant differences were observed between treatment (*p < 0.05 and **p < 0.01) by Student’s *t*-test.

To further investigate the function of *CsHMGB* in response to propamocarb stress, qRT-PCR was carried out to measure the quantity of *CsHMGB* transcripts within each organ. In “D0351” plants under propamocarb stress, the expression of *CsHMGB* was higher in the fruit peels and in the leaves than that in female flowers, moderate in the stems and fruit pulps, and weak in male flowers and in the roots (Figure 3A). *CsHMGB* expression was higher in “D0351” leaves and fruit peels than that in “D9320” leaves and fruit peels (Figure 3B). There were significant differences between “D0351” and “D9320” in *CsHMGB* mRNA abundance in fruit peels following propamocarb stress for 3, 6, 12, 24, 36, 48, 72, and 120 h. *CsHMGB* expression rapidly increased from 3 to 48 h and was significantly higher in “D0351” plants than in the water control treatment. However, from 72 to 120 h of propamocarb treatment, the expression of *CsHMGB* was reduced (Figure 3C). In “D9320” under propamocarb stress, the expression of *CsHMGB* displayed minimal change as compared to the control from 3 to 120 h (Figure 3D). In general, *CsHMGB* expression was positively correlated with propamocarb residues in the cucumber fruit peels.

### *CsHMGB* Is a Nuclear Protein

**FIGURE 4:**
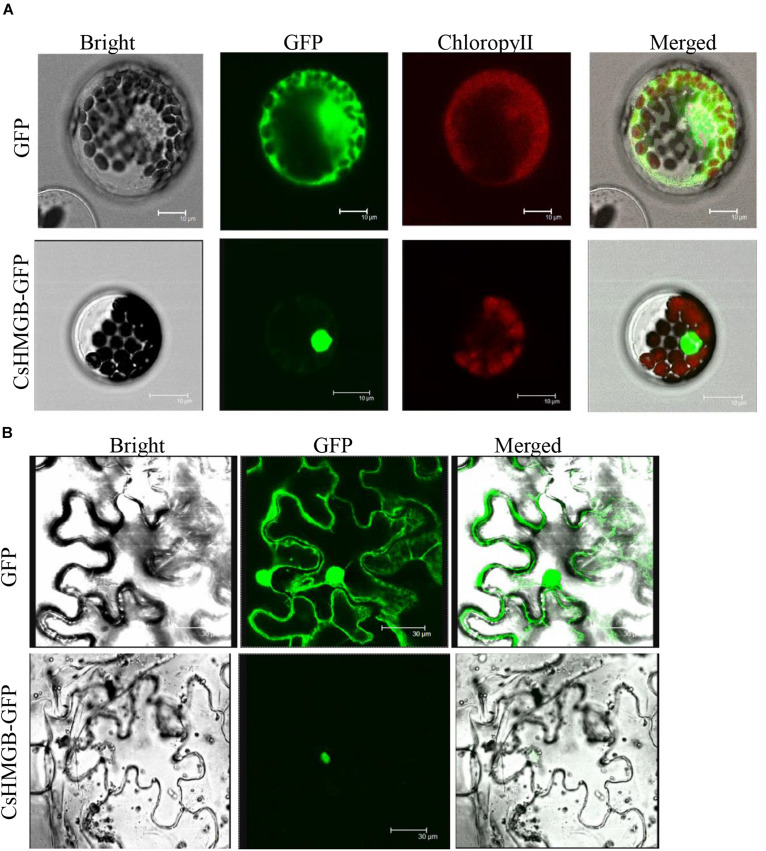
Subcellular localization of *CsHMGB* protein. **(A)** Subcellular localization of the *CsHMGB* protein in *Arabidopsis* protoplasts. The target protein of CsHMGB-GFP and empty plasmids (GFP) were observed with four channels of white light, UV light, and red light to localize the proteins. Bars of 10 μm. **(B)** Subcellular localization of the *CsHMGB* protein in leaf epidermis of *N. benthamiana*. Bars of 30 μm.

To determine the subcellular localization of *CsHMGB*, the CsHMGB-GFP protein was constructed and driven by the pSuper promoter. The pSuper, GFP empty vector, and CsHMGB-GFP fusion expression vector were transfected into *Arabidopsis* protoplast and *N. benthamiana* leaf epidermis. Using laser confocal microscopy, we determined that the GFP fluorescence signals were enriched in the nucleus in *Arabidopsis* protoplasts and in the *N. benthamiana* leaf epidermis (Figures 4A,B), indicating that *CsHMGB* is a nuclear protein.

### Silencing *CsHMGB* Results in Accumulation of More Propamocarb Residues in Cucumber

**FIGURE 5:**
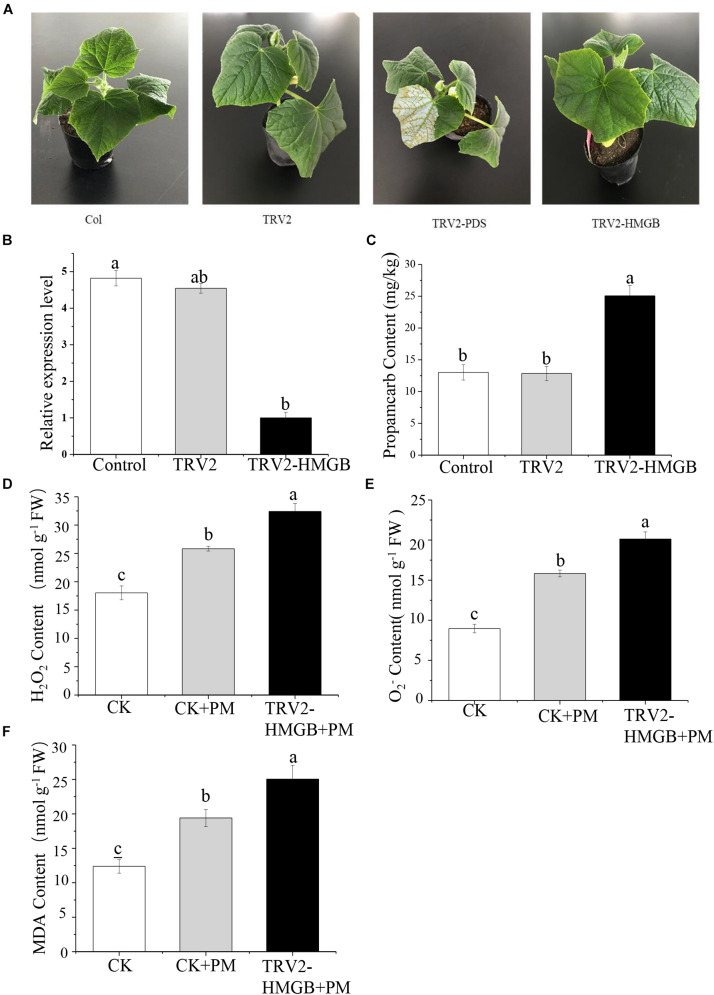
Silencing of CsHMGB in leaves of “D0351” plants. **(A)** Color fading phenotypes via PDS control gene 16 days after inoculation of leaves. **(B)** *CsHMGB* levels in *CsHMGB*-silenced, pTRV2 and control plants. **(C)** Propamocarb contents in leaves of *CsHMGB*-silenced, pTRV2, and control plants at 2 days. **(D)** O_2_^–^ contents in leaves under propamocarb stress. **(E)** H_2_O_2_ contents in leaves under propamocarb stress. **(F)** MDA contents in leaves under propamocarb stress. Different letters indicate significant differences between genotypes (*p* < 0.05 by Tukey’s test). Error bars represent SE.

To verify the function of the *CsHMGB* gene in response to propamocarb stress, the VIGS system was used to silence *CsHMGB* mRNA expression in “D0351” cucumber leaves. pTRV2, TRV2-PDS, TRV2-HMGB, and pTRV1 were mixed, and the mixture was applied to “D0351” cucumber rough leaves according to the *Agrobacterium tumefaciens* strain method. PDS was included as an indicator gene, and the leaves displayed photobleaching at 16 days after inoculation, indicating that *CsHMGB* was momentarily silenced (Figure 5A). The mRNA level of *CsHMGB* was markedly lower within *CsHMGB*-silenced leaves than within the TRV2 empty vector group and the control group (Figure 5B). Gas chromatography-tandem mass spectrometry revealed propamocarb residues in the control group and TRV2 group (14.94 and 13.85 mg/kg, respectively). However, *CsHMGB*-silenced leaves contained twice accumulations of propamocarb that of the control group (Figure 5C). These data demonstrate that *CsHMGB* plays a positive role in response to propamocarb stress.

In the control plants, we observed that the contents of O_2_^–^, H_2_O_2_, and MDA were increased in leaves under propamocarb stress, indicating that propamocarb may cause damage to cucumber plants though the accumulation of reactive oxygen species (ROS). In *CsHMGB*-silenced plants, O_2_^–^ and H_2_O_2_ contents increased by 127 and 125%, respectively, in comparison with the control after 48 h of propamocarb stress (Figures 5D,E). Similarly, MDA content was higher in *CsHMGB*-silenced plants than in control plants when under propamocarb stress (Figure 5F). These results indicate that resistance to propamocarb was reduced in *CsHMGB*-silenced plants under propamocarb stress.

### Activities of Antioxidant Enzymes in *CsHMGB*-Silenced Cucumber Plants

**FIGURE 6:**
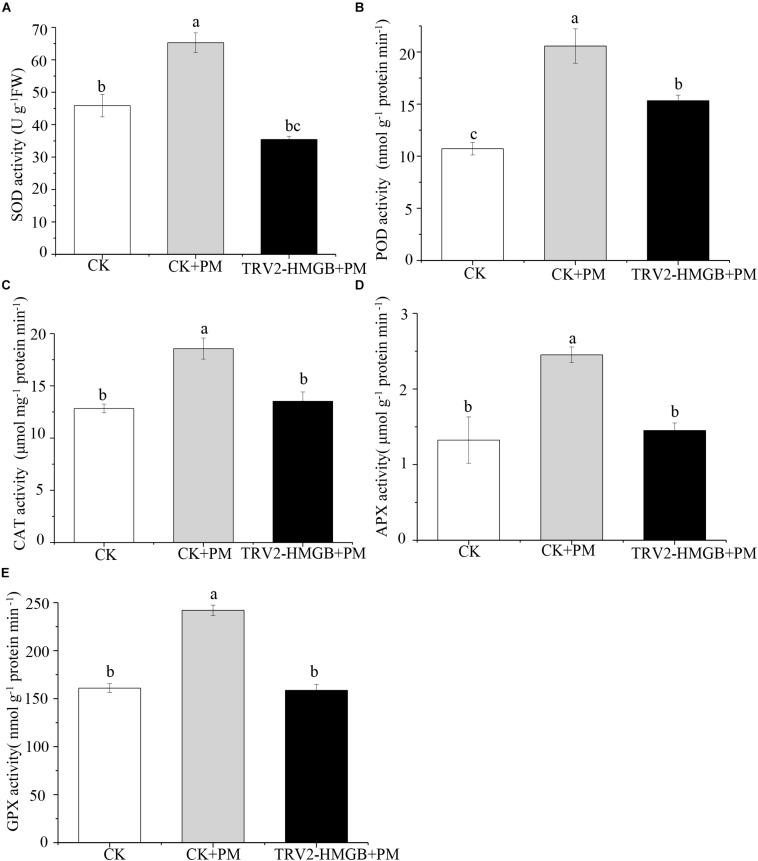
Activity of antioxidant enzymes in *CsHMGB*-silenced leaves of “D0351” plants. **(A)** SOD activity. **(B)** POD activity. **(C)** CAT activity. **(D)** APX activity. **(E)** GPX activity. Data are the means of three replicates with SEs, and different letters indicate a significant difference (*p* < 0.05 by Tukey’s test).

**FIGURE 7:**
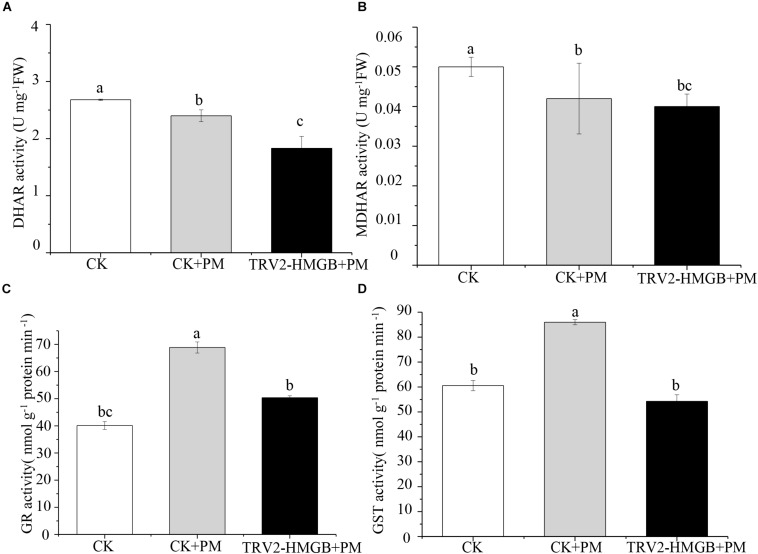
Activity of key enzymes in AsA-GSH system in *CsHMGB*-silenced leaves of “D0351” plants. **(A)** DHAR activity. **(B)** MDHAR activity. **(C)** GST activity. **(D)** GR activity. Data are the means of three replicates with SEs, and different letters indicate a significant difference (*p* < 0.05 by Tukey’s test).

The activity levels of antioxidant enzymes were associated with ROS-scavenging. As compared to the water control treatment, the activities of POD (Figure 6A), SOD (Figure 6B), CAT (Figure 6C), APX (Figure 6D), and GPX (Figure 6E) were enhanced under propamocarb stress. However, the activities of these enzymes were decreased in the *CsHMGB*-silenced plants. Notably, we observed reductions in the activities of detoxification-related enzymes in the ascorbate-glutathione (AsA-GSH) system, such as DHAR (Figure 7A), MDHAR (Figure 7B), GR (Figure 7C), and GST (Figure 7D) in the *CsHMGB*-silenced plants under propamocarb stress, as compared to control plants under propamocarb stress. The exchange of ascorbate from DHA to AsA and the regeneration of glutathione were inhibited by the silencing of *CsHMGB*, resulting in the decreased of the redox state of cells. As shown in Table 1, AsA, DHA, and total ascorbate contents decreased in *CsHMGB*-silenced plants under propamocarb stress compared to the control treatment with water, as did the redox state of the ascorbate (AsA/DHA). These changes suggested that silencing of *CsHMGB* promoted the conversion of ascorbate from AsA to DHA, leading to the decreased of the redox state of the ascorbate. Total glutathione content did not differ between the control and the *CsHMGB*-silenced plants; however, the ratio of GSH/GSSG was lower in the *CsHMGB*-silenced plants than in the control, indicating that the cucumber plants accelerated GSH consumption in response to propamocarb stress (Table 1). Taken together, these results strongly indicate that silencing of *CsHMGB* reduced antioxidation and detoxification in cucumber plants.

**TABLE 1:** The ASA-GSH system in *CsHMGB*-silenced leaves from “D0351” genotype under propamocarb stress at 4 days.

Lines	ASA content	DHA content	Total ascorbate	ASA/DHA	GSH content	GSSG content	Total glutathione	GSH/GSSG
Control	0.86^a^	1.32^a^	2.18^a^	0.65^a^	0.32^a^	0.23^b^	0.55^a^	1.39^a^
Control + PM	0.52^b^	0.92^b^	1.44^b^	0.57^b^	0.24^b^	0.33^a^	0.57^a^	0.73^b^
Slienced-HMGB	0.45^b,c^	0.78^c^	1.23^b,c^	0.58^b^	0.21^b^	0.34^a^	0.55^a^	0.62^b,c^

*The levels of total glutathione, DHA, ASA, GSH, total ascorbate, and GSSG are presented in the form of mmol g^–1^ FW. The redox states for ascorbate and GSH were determined, respectively, to be the ASA/DHA and GSH/GSSG ratios. Different letters indicate significant differences between genotypes (*p* < 0.05 by Tukey’s test). Error bars represent SE.*

### Overexpression of *CsHMGB* Alleviates Propamocarb Residues and Phytotoxicity in Cucumber

The overexpressed-vector of *CsHMGB*-PCXSN for transgenic experiments in cucumbers was constructed to assess the biological function of *CsHMGB* under propamocarb stress in cucumber plants, with an empty plasmid as a control group with the 35S promoter. Fifteen *CsHMGB*-overexpressed “D9320” cucumber plants and two empty “D9320” transgenic cucumber plants (as a control) were identified by qPCR through genetic transformation. Three OE lines, which shared the most abundant contents of *CsHMGB* (lines 2, 3, and 5), were harvested from the T_1_ generation after self-pollination (Supplementary Figure S3).

**FIGURE 8:**
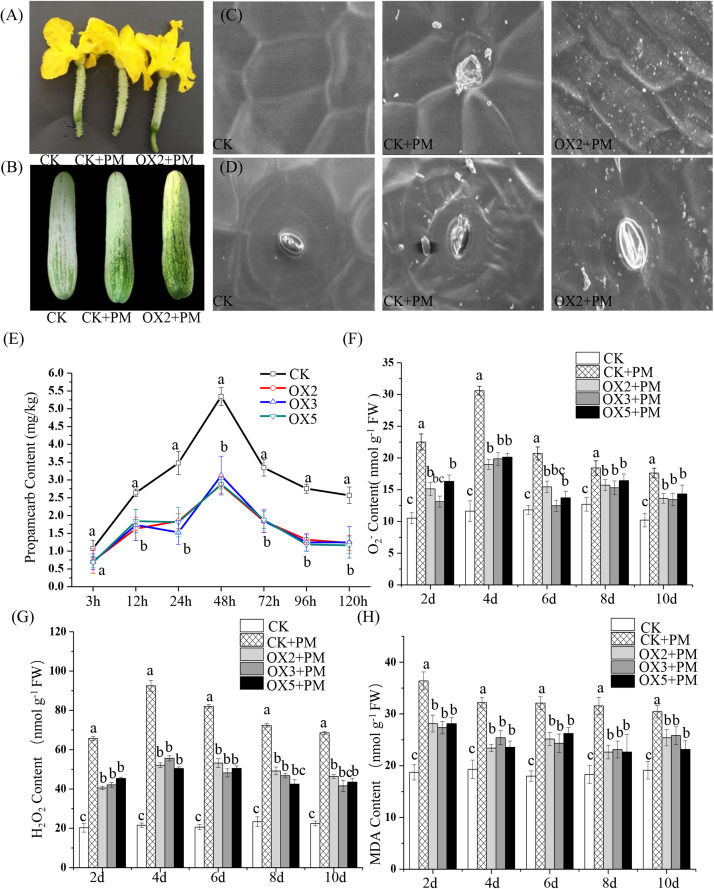
Effects of *CsHMGB* on propamocarb residues and phytotoxicity in “D9320” plants. **(A)** Phenotypes of fruits from the control (Col) and *CsHMGB* overexpressing (OX) plants at 4 days post anthesis (DPA). **(B)** Phenotypes of fruits from Col and OX plants at 12 DPA. **(C)** The accumulation of waxiness on epidermal cells in fruit peels. Bars of 20 μm. **(D)** The opening of the stomatal aperture in fruit peel. Bars of 20 μm. **(E)** Propamocarb contents in fruit peels under propamocarb stress with time course of 3, 12, 24, 48, 72, 96, and 120 h. **(F–H)** O_2_^–^, H_2_O_2_, and MDA contents in leaves under propamocarb stress with time course of 2, 4, 6, 8, and 10 days. Data are the means of three replicates with SEs, and different letters indicate a significant difference (*p* < 0.05 by Tukey’s test).

We sprayed 400 ppm propamocarb on the *CsHMGB*-overexpressed plants and the control plants. We observed no phenotypic differences (such as fruit length and color of fruit peel) between the *CsHMGB*-overexpressed plants and the control plants (Figures 8A,B). However, anatomical analysis of fruit peels under a scanning electron microscope revealed distinct differences. With the stomatal opening under the water treatment as a control, the observed increase of the stomatal aperture was larger in *CsHMGB*-overexpressed plants as compared to control plants under propamocarb stress (Figure 8D). Waxiness in the fruit peels was notably greater in *CsHMGB*-overexpressed plants, while minimal waxiness was noted in the control plants following application of propamocarb (Figure 8C). Changes in propamocarb residues were analogous between *CsHMGB*-overexpressed and control plants, with both increasing from 3 to 48 h and decreasing after 48 h. Residue levels in the fruit peels were lower in *CsHMGB*-overexpressed plants than in the control, and decreased by 48.7, 41.0, and 42.1% in the OX2, OX 3, and OX 5 lines at 48 h (Figure 8E), respectively. These findings indicate that overexpression of *CsHMGB* alleviates propamocarb residue accumulation through increasing waxiness and the stomatal conductance.

Under propamocarb stress conditions, O_2_^–^ and H_2_O_2_ contents increased from 2 to 4 days then decreased around 4–10 days in both *CsHMGB*-overexpressed and control plants. O_2_^–^ and H_2_O_2_ contents were lower in *CsHMGB*-overexpressed plants than in control plants (Figures 8F–G). MDA content also decreased in *CsHMGB*-overexpressed plants under propamocarb stress (Figure 8H). These results indicate that overexpression of *CsHMGB* enhances the resistance of cucumber plants to propamocarb stress.

### Overexpression of *CsHMGB* Enhances the Activities of Antioxidant Enzymes

**FIGURE 9:**
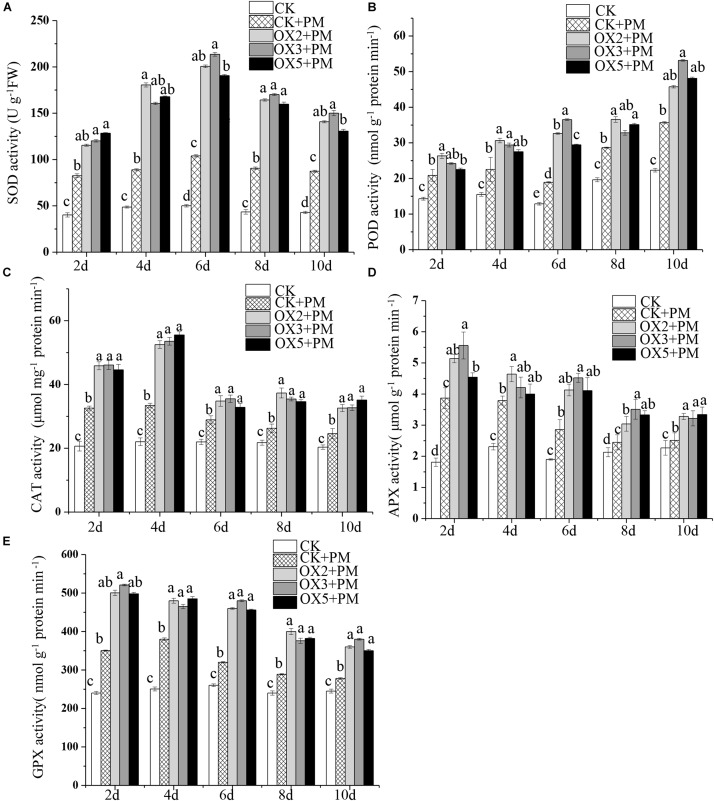
Activity of antioxidant enzymes in *CsHMGB*-overexpressing (OX) leaves of “D9320” plants. **(A)** SOD activity. **(B)** POD activity. **(C)** CAT activity. **(D)** APX activity. **(E)** GPX activity. Data are the means of three replicates with SEs, and different letters indicate a significant difference (*p* < 0.05 by Tukey’s test).

**FIGURE 10:**
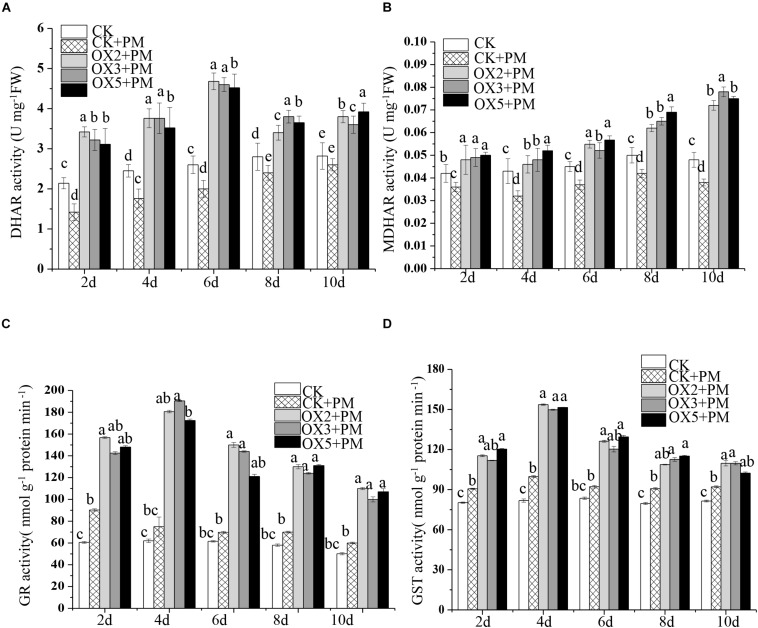
Activity of key enzymes in AsA-GSH system in *CsHMGB*-overexpressing (OX) leaves of “D9320” plants. **(A)** DHAR activity. **(B)** MDHAR activity. **(C)** GST activity. **(D)** GR activity. Data are the means of three replicates with SEs, and different letters indicate a significant difference (*p* < 0.05 by Tukey’s test).

Compared to the control, the activities of SOD (Figure 9A), CAT (Figure 9C), APX (Figure 9D), and GPX (Figure 9E) were increased from 2 to 6 days and then decreased from 6 to 10 days in the *CsHMGB*-overexpressed plants under propamocarb stress. The activity of POD (Figure 9B) was more enhanced from 2 to 10 days in the OX2, OX 3, and OX 5 lines than in the control. As shown in Table 2, the AsA, DHA, and total ascorbate contents, as well as the redox state of ascorbate (AsA/DHA), were enhanced in the OX2, OX3, and OX5 lines as compared to the control. *CsHMGB* accelerated the transformation of DHA to AsA and improved the antioxidant capacity (Table 2). In a surprising finding, there were no notable differences in GSSG, GSH, or total glutathione contents between the *CsHMGB*-overexpressing lines and the control lines under propamocarb treatments. However, the redox state of GSH/GSSG was greater in *CsHMGB*-overexpressing lines as compared to the control lines. It is important to note that overexpression of *CsHMGB* increased the activities of DHAR (Figure 10A), MDHAR (Figure 10B), GST (Figure 10C), and GR (Figure 10D). *CsHMGB*-overexpression significantly induced the accumulation of total ascorbate and glutathione following with the exchange of ascorbate from DHA to AsA and the regeneration of glutathione, leading to an enhanced redox state of cells. These findings indicate that overexpression of *CsHMGB* increases key enzymatic activities of AsA-GSH system and promotes propamocarb metabolism in cucumber plants.

**TABLE 2:** The ASA-GSH system in *CsHMGB*-overexpressing leaves from “D9320” genotype under propamocarb stress at 4 days.

Lines	ASA content	DHA content	Total ascorbate	ASA/DHA	GSH content	GSSG content	Total glutathione	GSH/GSSG
CK	1.22^c^	1.80^c^	3.02^d^	0.68^b^	0.42^d^	0.33^a^	0.75^d^	1.27^e^
CK + PM	1.61^b^	2.80^b^	4.41^c^	0.58^c^	0.62^c^	0.22^b^	0.84^c^	2.82^d^
OX2 + PM	2.45^a^	3.19^a^	5.64^b^	0.77^a^	0.77^b^	0.19^c^	0.96^b^	4.05^b^
OX3 + PM	2.78^a^	3.77^a^	6.55^a^	0.74^a^	0.82^a^	0.21^b^	1.03^a^	3.90^c^
OX5 + PM	2.62^a^	3.58^a^	6.20^a^	0.73^a^	0.86^a^	0.17^c^	1.03^a^	5.06^a^

*The levels of total glutathione, DHA, ASA, GSH, total ascorbate, and GSSG are presented in the form of mmol g^–1^ FW. The redox states for ascorbate and GSH were determined, respectively, to be the ASA/DHA and GSH/GSSG ratios. Different letters indicate significant differences between genotypes (*p* < 0.05 by Tukey’s test). Error bars represent SE.*

### Overexpression of *CsHMGB* Induces Increased Key Genes Expression in AsA-GSH System

**FIGURE 11:**
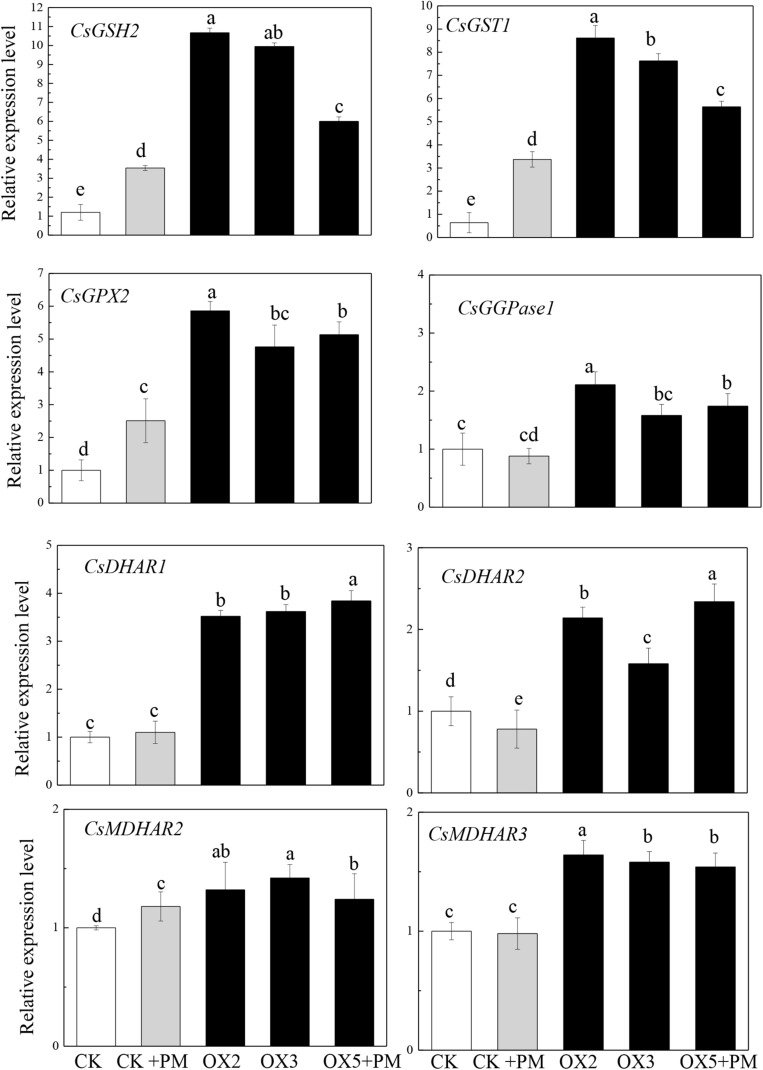
Expression levels of key genes in in AsA-GSH system between the control (Col) and *CsHMGB*-overexpressing (OX) plants. *CsEF1a* was used as the reference gene. Different letters indicate significant differences between genotypes (*p* < 0.05 by Tukey’s test). Error bars represent SE.

To investigate the effects of *CsHMGB* on the transcript levels of genes encoding antioxidant enzymes in AsA-GSH, qRT-PCR was carried out to determine the expression levels of key genes. As shown in Figure 11, *CsGPX2*, *CsGST1*, and *CsGSH2 CsMDHAR2* were induced by propamocarb stress, *CsGGPase1*, *CsDHAR1*, *CsDHAR2* and *CsMDHAR3* were inhibited by propamocarb stress. Additionally, these genes were significantly up-regulated in the OX2, OX3, and OX5 lines as compared to the control. This result shows that *CsHMGB* induces increased key genes expression in AsA-GSH system.

## Discussion

Analyses of mRNA expression and protein localization aid in determining gene functions. A previous study suggested that *CsABCA19*, a membrane transporter protein, was markedly increased in the fruit peels of “D0351” plants under propamocarb stress, but found little change in “D9320” plants (Meng et al., 2016). In addition, mitochondrial proteins of *CsMCF* and glutathione proteins of *CsMAPEG* are up-regulated in the leaves and fruits of propamocarb-stressed “D0351” plants (Zhang et al., 2019a, b). Our results showed that the expression pattern of *CsHMGB* was consistent with those of *CsABCA19*, *CsMCF*, and *CsMAPEG*, which are positively correlated with propamocarb residues in the fruit peels of “D0351” plants and up-regulated in leaves and fruit peels. This indicates that *CsHMGB* is up-regulated in response to propamocarb stress. Eight HMGB proteins have been identified in *A. thaliana*; AtHMGB1, AtHMGB5, AtHMGB6, and AtHMGB14 are primarily located in the nucleus, whereas AtHMGB2 and AtHMGB3 have been reported in both the nucleus and the cytoplasm (Launholt et al., 2006; Grasser et al., 2007; Lildballe et al., 2008; Pedersen et al., 2010). Although AtHMGB12 is unable to bind to DNA, it enables interaction with XPO1a (the nuclear output receptor) and is located in the cytoplasm (Štros et al., 2007). OsHMGB is an OsCML3 target protein, and binds to DNA within the nucleus to regulate intracellular Ca^2+^ (Aumnart et al., 2015). Several studies have shown that HMGB proteins are localized to the nucleus, are able to bind to specific DNA structures, and are involved in a variety of important biological processes (Grasser et al., 2007; Kang et al., 2014). In our study, six HMGB (*CsHMGB* and *CsHMGB*1/2/3/6/14) proteins were identified in cucumbers, and cluster analysis showed that the HMGB proteins were contained in one branch between cucumber and *A. thaliana* (Supplementary Figure S4). *CsHMGB* (*CsaV3_5G021890.1*) shared the highest sequence similarity with AtHMGB5 (*AT4G35570.1*) was screened for gene function under propamocarb stress. We found that *CsHMGB* was localized to the nucleus (Figures 4A,B), indicating that *CsHMGB* might have a similar function to transcription factors and may specifically bind to DNA or chromatin in response to propamocarb stress.

Several studies have demonstrated that HMGBs play an important regulatory role in responses to abiotic stress (Reeves, 2010). Heterologous expression of *CsHMGB* in *A. thaliana* influences the germination of seeds in the presence of drought and high-salt stress (Jang et al., 2008). In addition, *AtHMGB2* and *AtHMGB3* expression levels are markedly decreased under salt and drought stress. Overexpression of *AtHMGB1* and *AtHMG2* decreases seed germination and inhibits seed growth in the presence of salt stress, and *AtHMGB2*, *AtHMGB3*, and *AtHMGB4* are up-regulated under cold stress (Kwak et al., 2007; Lildballe et al., 2008). In rice, transcriptome data showed that *OsHMGB* was rapidly up-regulated under cold treatment (Yang et al., 2015) and the functional deletion mutant *Oshmgb710* demonstrated high susceptibility to rice bacterial blight; lines overexpressing *OsHMGB710* were significantly more resistant to bacterial blight (Ju, 2017). HMGB1 is also a marker of non-specific and sensitive inflammation in mammals, including humans. HMGB1 can induce the release of cytokines and participate in a variety of pathophysiological processes in higher animals (Harris et al., 2012; Venereau et al., 2016; Mc et al., 2019). In the lungs of rats, HMGB1 abundance was shown to be beneficial following paraquat poisoning. HMGB1 is involved in pathophysiological reactions, and changes in its expression reflect the progression of paraquat poisoning (Ju and Zhang, 2019). In the present study, silencing of *CsHMGB* increased the accumulation of propamocarb residues in cucumber plants (Figure 5C), while propamocarb residues were significantly decreased in *CsHMGB*-overexpressing cucumber plants (Figure 8E). These findings show that *CsHMGB* plays a crucial role in regulating propamocarb residues.

Waxiness of the plant epidermis provides a protective barrier that inhibits non-stomatal water loss and protects plants from diseases and insect pests. In *Arabidopsis*, bryophytes, and soybeans, increased wax content is correlated with drought resistance (Thoenes et al., 2004; Buda et al., 2013; Luo et al., 2013). In cucumbers, the *CsCER1* and *CsWAX2* biosynthesis genes respond to drought stress, light intensity, and other external adverse environmental factors by inducing the accumulation of pericarp wax (Liu et al., 2014; Wang et al., 2015a,b). Notably, stomatal action played a crucial role in the response to plant stress. Low temperature induced increased NO accumulation and decreased stomatal conductance in cucumber plants (Zhang Z. W. et al., 2019). In tomatoes, rapid and increased stomatal closure was co-related with drought tolerance in the *SlWRKY81*-silenced plants (Ahammed et al., 2019). Here, we found that increased wax content and stomatal conductance alleviated propamocarb residue levels in the *CsHMGB*-overexpressed plants (Figures 8B,C). These findings show that *CsHMGB* promotes the self-protective mechanisms and propamocarb metabolism in cucumber.

Antioxidant defense plays a crucial role in plant abiotic stress. In cucumber, melatonin has been shown to alleviate iron stress by improving antioxidant defense and reducing the accumulation of ROS (Ahammed et al., 2020b). In tomato, melatonin is essential for maintaining the proper antioxidant potential, leading to amelioration of Cd-induced oxidative stress (Hasan et al., 2019). Dopamine alleviated bisphenol A-induced phytotoxicity (an emerging organic pollutant) by enhancing antioxidant and detoxification potential in cucumber (Ahammed et al., 2020a). Silicon improved the activity of antioxidant enzymes and decreased MDA and ROS levels in response to low levels of phosphorus stress in tomato plants (Zhang Y. et al., 2019). Overexpression of *CsMCF* in the presence of propamocarb in cucumbers resulted in enhanced antioxidant enzyme activity and reduced lipid peroxidation levels (Zhang et al., 2019a). Similarly, in the present study, the enhancement of antioxidant enzyme activity induced by overexpressing of *CsHMGB* was also confirmed to involve in detoxification of ROS produced under propamocarb stress in cucumber.

Ascorbate-glutathione cycle acts as an effective antioxidant system for the detoxification of excess ROS and maintaining the redox state of plant cells (Bartoli et al., 2017). Under propamocarb stress, the increasing activity and transcript levels of DHAR and MDHAR positively promoted the AsA regeneration, resulting in the enhancement of the ratio of AsA/DHA and total ascorbic content. Furthermore, the expression of key genes in the glutathione metabolic pathway, *CsGPX2*, *CsGSH2*, and *CsGST1* were induced by overexpression of *CsHMGB* under propamocarb stress, together with the increased activity of GR and GST, accordingly, both the ratio of GSH/GSSG and total glutathione content were significantly increased. These changes contributed to an enhanced redox state of the ascorbate and glutathione for ROS scavenging and propamocarb detoxification (Figures 9–11 and Table 2). In tomatoes, overexpression of CAFFEICACID OMETHYLTRANSFERASE 1 (*COMT1*) and exogenous melatonin alleviated carbendazim phytotoxicity and pesticide residue levels by improving the activity of antioxidant enzymes and activation of the AsA-GSH system and reduction of ROS and MDA levels (Yan et al., 2019). In wheat, the enhanced AsA-GSH system and antioxidant enzyme activities alleviated Cd stress (Qin et al., 2018). Notably, glutathione plays an important role in the detoxification of pesticide residues in plants (Yu et al., 2013). Application of brassinosteroids and silencing of respiratory burst oxidase-1 (*RBOH1*) resulted in H_2_O_2_-dependent redox homeostasis, glutathione biosynthesis, and increased GST activity to limit CHT residue levels in tomatoes (Zhou et al., 2015). Thus, *CsHMGB* alleviates phytotoxicity and propamocarb residue levels by improving antioxidant potential, ascorbic and glutathione-dependent detoxification.

## Conclusion

**FIGURE 12:**
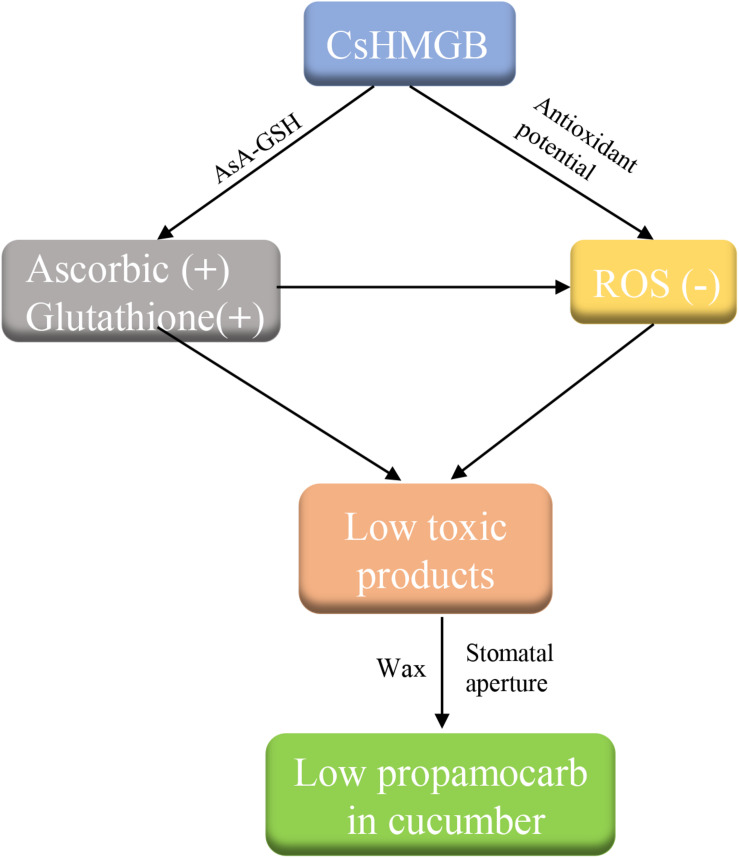
Simple pathway model of propamocarb degradation and metabolism in cucumbers.

We cloned a 624 bp of *CsHMGB* that contained the conserved HMB-box region. *CsHMGB* expression was positively correlated with propamocarb residue levels in cucumber fruit peels. *CsHMGB* was upregulated in fruit peels of “D0351” genotype plants following exposure to propamocarb stress for 3–120 h. The CsHMGB protein was targeted to the nucleus. In CsHMGB-silenced plants, resistance was reduced and propamocarb residues were increased following increases in MDA content and ROS; reductions in antioxidant enzyme activity (SOD, POD, CAT, APX, GPX, GST, and GR) and the AsA-GSH system were also observed. Overexpression of *CsHMGB* promoted glutathione-dependent detoxification by AsA-GSH system and improved the antioxidant potential, reduced the accumulation of ROS. Ultimately, propamocarb metabolism in cucumber was increased via increase in the wax levels and the stomatal conductance (Figure 12). Thus, *CsHMGB* contributes to lower propamocarb residue levels, with possible applications in the production and breeding of cucumbers to achieve lower propamocarb residue levels in fruits.

## Data Availability Statement

The datasets generated for this study can be found in the http://www.icugi.org/.

## Author Contributions

SL and ZQ designed and conceived the research. MX, JL, CW, DL, WZ, and XZ performed the experiments. CW, DL, CL, and WZ analyzed the sequencing data. SL wrote the entire manuscript. ZQ and XZ edited the manuscript.

## Conflict of Interest

The authors declare that the research was conducted in the absence of any commercial or financial relationships that could be construed as a potential conflict of interest.

## Footnotes

1 http://www.icugi.org/

2 http://www.ncbi.nlm.nih.gov/nucleotide/

3 http://www.lynnon.com/

## Supplementary Material

FIGURE S1 Phenotypes of fruits in “D0351” and “D9320” genotype.

FIGURE S2 Sequence and phylogenetic analysis of *CsHMGB*. **(A)** Analysis of *CsHMGB* sequence. **(B)** Phylogenetic analysis of *CsHMGB* with HMGB proteins in other species; the amino acid sequences of the *CsHMGB* and HMGB proteins were used to construct phylogenetic trees using a neighbor-joining algorithm. Cp, *Cucurbita pelo*; At, *Arabidopsis thaliana*; Cs, *Cucumis sativus*; Pp, *Prunus persica*; Pa, *Populus alba*; Cm, *Cucumis melo*; Mc, *Momordica charantia;* Ns, *Nicotiana Sylvestris*; Nt, *Nicotiana tabacum*; Pt, *Populus trichocarpa*; Vv, *Vitis vinifera*. **(C)** Conservative HMGB-box structure in various species.

FIGURE S3 *CsHMGB* levels in the control (Col) and *CsHMGB* -overexpressing (OX) plants were screened by qPCR.

FIGURE S4 Phylogenetic tree of HMG proteins from cucumber (Cs) and Arabidopsis (At).

TABLE S1 Sequences of the oligo nucleotides used in this study.
