# Supplementary figures and images for: Overexpression of CsHMGB Alleviates Phytotoxicity and Propamocarb Residues in Cucumber
Source: Front Plant Sci. 2020 Jun 12;11:738. doi: 10.3389/fpls.2020.00738 (PMC7304447; doi:10.3389/fpls.2020.00738)

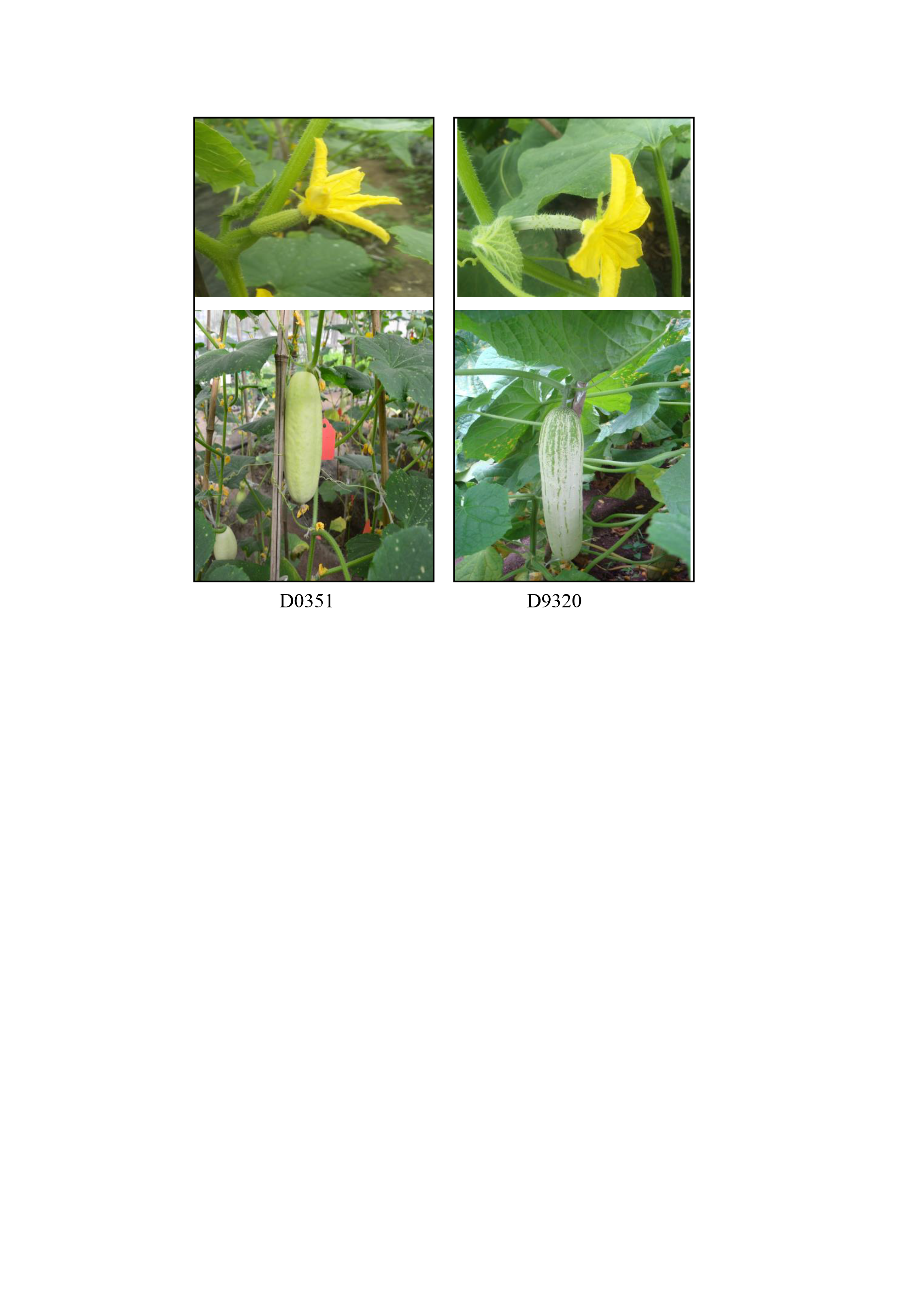

Supplement: FIGURE S1 — Phenotypes of fruits in “D0351” and “D9320” genotype. [file Image_1.TIF]

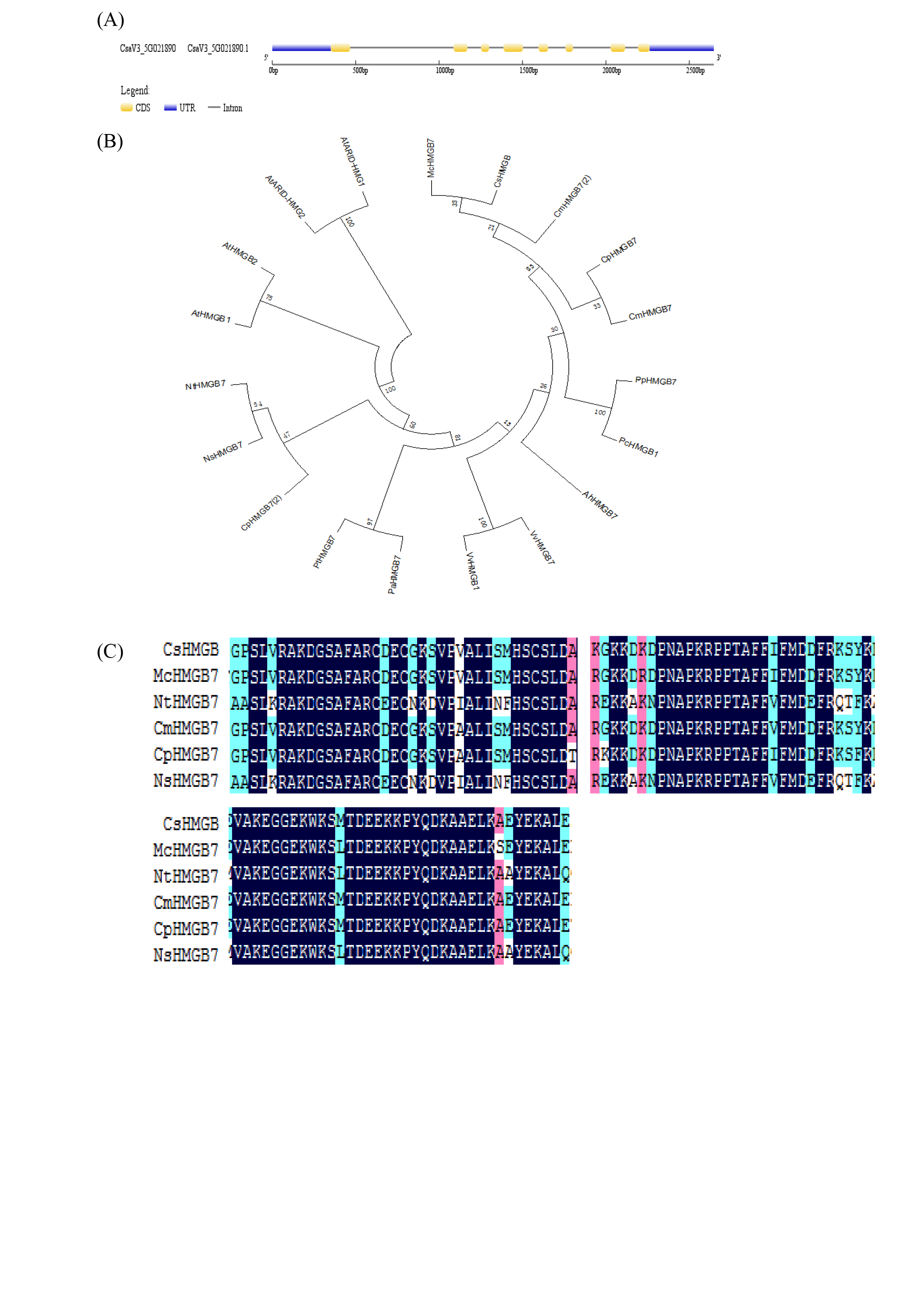

Supplement: FIGURE S2 — Sequence and phylogenetic analysis of CsHMGB. (A) Analysis of CsHMGB sequence. (B) Phylogenetic analysis of CsHMGB with HMGB proteins in other species; the amino acid sequences of the CsHMGB and HMGB proteins were used to construct phylogenetic trees using a neighbor-joining algorithm. Cp, Cucurbita pelo; At, Arabidopsis thaliana; Cs, Cucumis sativus; Pp, Prunus persica; Pa, Populus alba; Cm, Cucumis melo; Mc, Momordica charantia; Ns, Nicotiana Sylvestris; Nt, Nicotiana tabacum; Pt, Populus trichocarpa; Vv, Vitis vinifera. (C) Conservative HMGB-box structure in various species. [file Image_2.TIF]

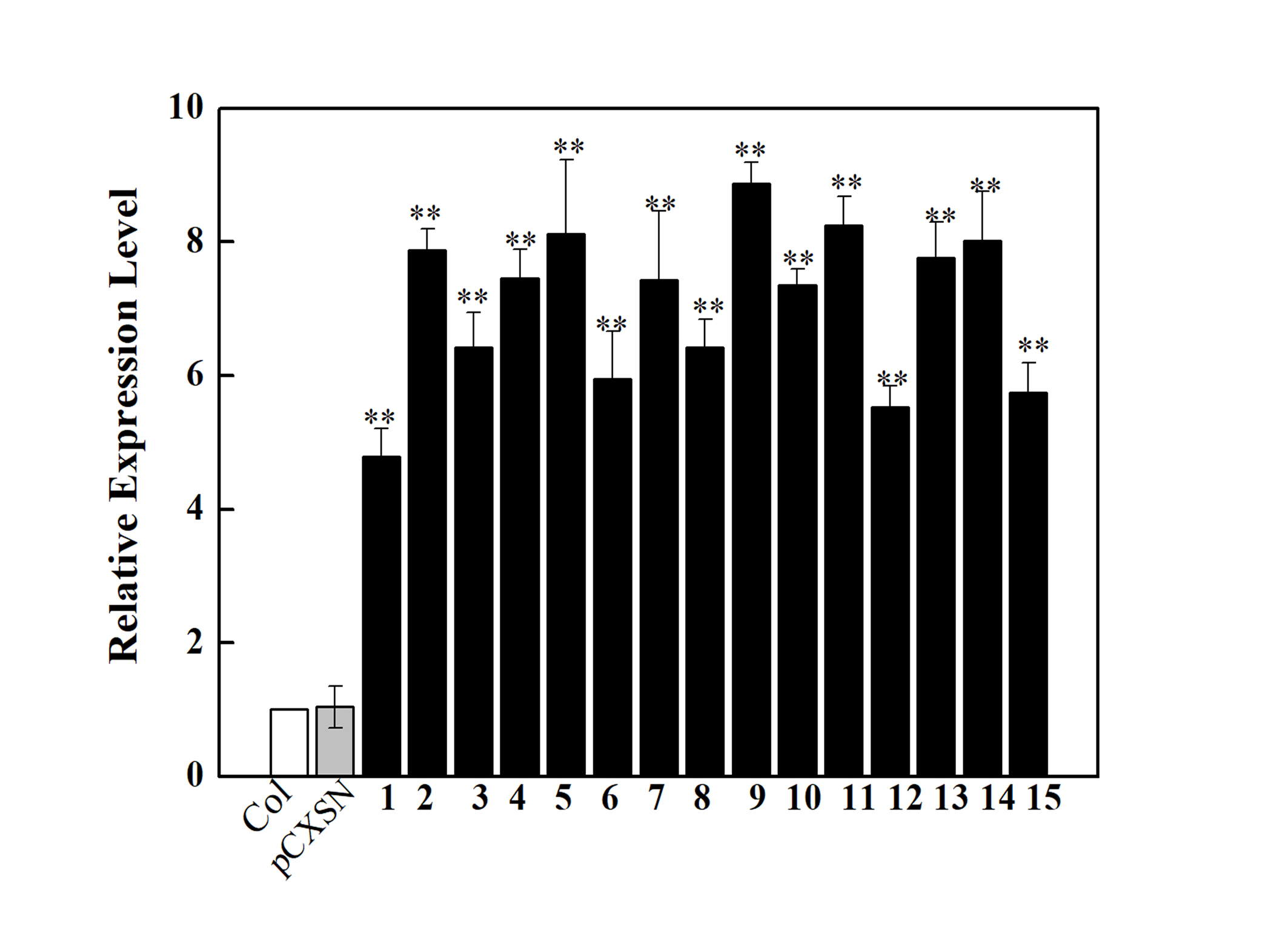

Supplement: FIGURE S3 — CsHMGB levels in the control (Col) and CsHMGB -overexpressing (OX) plants were screened by qPCR. [file Image_3.TIF]

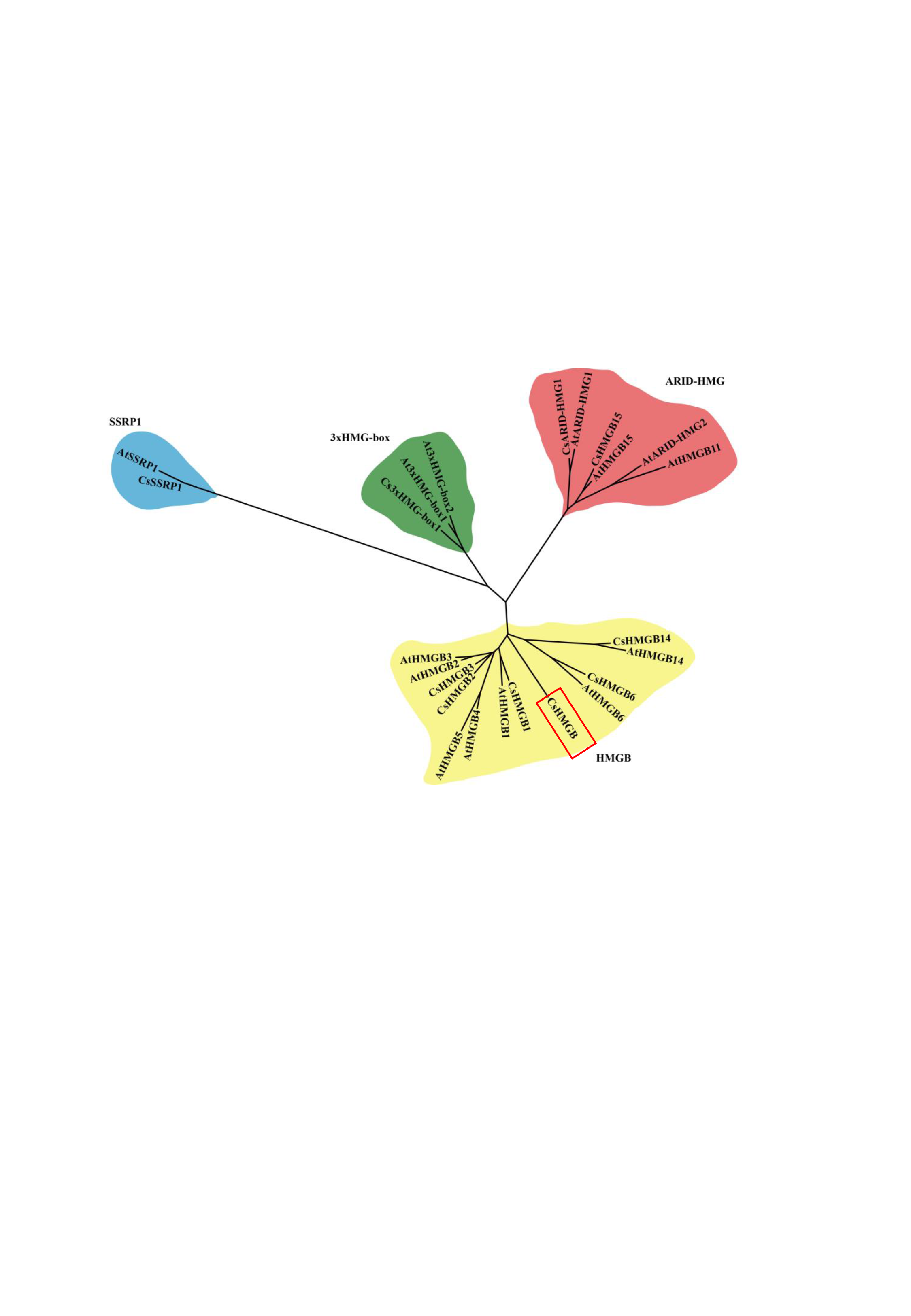

Supplement: FIGURE S4 — Phylogenetic tree of HMG proteins from cucumber (Cs) and Arabidopsis (At). [file Image_4.TIF]
