# Supplementary material for: Overexpression of CsHMGB Alleviates Phytotoxicity and Propamocarb Residues in Cucumber
Source: Front Plant Sci. 2020 Jun 12;11:738. doi: 10.3389/fpls.2020.00738 (PMC7304447; doi:10.3389/fpls.2020.00738)
Supplement: TABLE S1 — Sequences of the oligo nucleotides used in this study. [file Table_1.DOCX]

Table S1 **Sequences of the oligo nucleotides used in this study.**

| **Oligo name** | **Oligo sequence（5’**→ **3’）** | **Description** | **Gene ID** |
| --- | --- | --- | --- |
| HMGB-F | CTCCAAGTCCCGGAAGAGAG | qRT-PCR analysis | CsaV3-5G28190 |
| HMGB-R | GATTAAGGCCACCGGAACAC |  |  |
| CsGSH2-F | AATCCTTGAGAACAATGGCG | qRT-PCR analysis | CsaV3-1G039210 |
| CsGSH2-R | GGTGAATGAGACAAACCGACT |  |  |
| CsGST1-F | ATGCATCGGCTTCACAAGAC | qRT-PCR analysis | Csa4G280630.1 |
| CsGST1-R | CCGATCACAGGCATCTCTCT |  |  |
| CsGPX2-F | TCTGAAGCAAGAGCCTGGAA | qRT-PCR analysis | CsaV3_7G028610 |
| CsGPX2-R | GGAGCCTATGAACCCATTGC |  |  |
| CsDHAR1-F | CTTCTGTGGGATCGAAGATATTTAG | qRT-PCR analysis | Csa5G179760.1 |
| CsDHAR1-R | CATGGTACAACTTCGGTGCTG |  |  |
| CsDHAR2-F  CsDHAR2-R | CTCCTGACAAATCTTCAGTAGGATC  CAACTTTGGACCAAGTGACAGAT | qRT-PCR analysis | Csa3G285550.1 |
| CsMDHAR2-F  CsMDHAR2-R | TGGAGTGGCAGCAGGATATG  GAGGATCAATTCTATCCCTTTCTCTTC | qRT-PCR analysis | Csa3G099720.1 |
| CsMDHAR3-F  CsMDHAR3-R | TGAAGCACAGTGCATGCC  CCAACAACAACCATGTCAACA | qRT-PCR analysis | Csa5G524740.1 |
| CsGGpase1-F  CsGGpase1-R | TGGTTTCATTGCCCAGCT  GGTTGCCATTAATGTCAGC | qRT-PCR analysis | CsaV3-7G02202 |
| CsHMGB-F | ATGGCCGGCGGAGGATCCTC | PCR clone | CsaV3-5G28190 |
| CsHMGB-R | TTACTCTTCTTCCGTTATTCC |  |  |
| CsHMGB-GF | CTCTTCTTCCGTTATATGGCCGGCG | Subcellular localization | CsaV3-5G28190 |
| CsHMGB-GR | GAAGATCTCTCTTCTTCCGTTAT |  |  |
| VIGS-HMGB-F | CGGAATTCTGCGGTAAAAGTGTTCCGG | Slience of gene | CsaV3-5G28190 |
| VIGS-HMGB-R | ATAAAGAAAGCTGTAGGAGATAAAGAAAGCTGTAGG |  |  |
| PCXSN-1250-F | CGGCAACAGGATTCAATCTTA | Transgenic detection |  |
| PCXSN-1250-R | CAAGCATTCTACTTCTATTGCAGC |  |  |
